# Evaluating the Adverse Events Associated with Three Doses of the COVID-19 Vaccination in Adults in the Western Region of Saudi Arabia: A Cross-Sectional Study

**DOI:** 10.3390/vaccines11020266

**Published:** 2023-01-26

**Authors:** Hamzah J. Aldali, Jehad A. Aldali, Badi A. Alotaibi, Glowi A. Alasiri, Aroob M. Alromih, Emadeldin M. Elsokkary, Ali Z. Aldali, Abdullah Almeziny

**Affiliations:** 1 Cellular and Molecular Medicine, College of Biomedical Science, University of Bristol, Bristol BS8 1TD, UK; 2 0921 Department of Pathology, Faculty of Medicine, Imam Mohammad Ibn Saud Islamic University, Riyadh 13317, Saudi Arabia; 3 Department of Clinical Laboratory Sciences, Collage of Applied Medical Sciences, King Saud Bin Abdulaziz University for Health Sciences, Riyadh 11481, Saudi Arabia; 4 King Abdullah International Medical Research Center, Riyadh P.O. Box 3660, Saudi Arabia; 5 Department of Biochemistry, College of Medicine Organization Imam Mohammad Ibn Saud Islamic University, Riyadh 13317, Saudi Arabia; 6 Medical School, Imam Mohammad Ibn Saud Islamic University, Riyadh 13317, Saudi Arabia; 7 Psychology, Organisation Imam Mohammad Ibn Saud Islamic University, Riyadh 13317, Saudi Arabia; 8 Department of Rehabilitation Health Science—Physical Therapy, College of Applied Medical Sciences, King Saud University, Riyadh 11445, Saudi Arabia

**Keywords:** vaccines, COVID-19, coronavirus, infectious diseases, adverse events

## Abstract

The Kingdom of Saudi Arabia was one of the countries earliest affected by the coronavirus 2019 (COVID-19) pandemic and had taken precautions including compulsory COVID-19 vaccination. Both the ChAdOx1 nCoV-19 vaccine (Oxford AstraZeneca) and the BNT162b2 vaccine (Pfizer) were approved by the Saudi Ministry of Health, followed by mRNA-1273 (Moderna), all of which were used for population-wide vaccination. This study aimed to assess the short-term side effects following the COVID-19 vaccinations among participants who had received all three doses in the western region of Saudi Arabia. An online survey was distributed to the participants who received either BNT162b2, ChAdOx1 nCoV-19, or mRNA-1273 vaccines, and the type of side effects and their severity were evaluated. Fatigue and headache, pain at the site of the injection and muscle pain were the most common side effects in all three doses. However, the severity depending on the type of vaccination was significant only for the first and second dose, but not the third dose. In contrast, there was a higher percentage of participants who encountered severe side effects from the third dose compared to the first and second. Nevertheless, the majority of participants described all three doses’ side effects to be moderately severe. A future evaluation could be made to access the individual types of vaccination and compare between the side effects of the BNT162b2, ChAdOx1 nCoV-19, and mRNA-1273 vaccines specifically for the booster dose.

## 1. Introduction

Despite many lockdowns and long-term infection control measures implemented in most countries, the 2019 coronavirus (COVID-19) pandemic, which started in China in December 2019, is still spreading [1,2,3]. Since the beginning of the COVID-19 pandemic, both hospital and community infection control efforts have been employed to minimize the risk of infection spread and some safe vaccines have been generated [4]. Several vaccination options have been accessible for use and found to be safe and effective. By the end of 2020, many countries, including Gulf countries, employed vaccination campaigns due to the promising effectiveness of the COVID-19 vaccines. However, all of the vaccines proposed by the World Health Organization do not provide complete immunization against the disease [4,5]. Therefore, administering more than one dose was proposed in many countries [6]. Comparing COVID-19 infections with seasonal influenza, it was found that the influenza vaccine contributed to lower rates of hospitalization and mortality, while the COVID-19 vaccination significantly reduced death and hospitalization rates in the elderly group only [7]. COVID-19 vaccination campaigns have been introduced to reach the public and private sectors in many countries. Furthermore, some countries made it mandatory to be vaccinated in order to enter the country [8]. The Kingdom of Saudi Arabia was one of the first countries to take precautionary actions including compulsory COVID-19 vaccination for the first, followed by the second and third dosage of vaccination [9,10]. More importantly, to lessen the impact of the disease, Saudi health officials implemented early, unprecedented preventive measures and precautionary strategies, including the cancellation of social events, prohibiting international flights, closing schools and universities, work-from-home mandates, curfews, and placing the entire nation under complete lockdown [5,9]. These approaches were incredibly effective. These initiatives did not include reliable test and contact tracing protocols. As a result, a second wave of the virus hit numerous Arab Gulf nations, prompting additional lockdowns [5]. However, in the absence of an approved antiviral treatment for COVID-19, several vaccine development studies were quickly launched in hopes of bringing the pandemic under control [6]. As a result, immunization programs started in developed countries and then expanded to other parts of the world, but the administrative problems of manufacturing, transporting, and managing billions of doses on a global scale presented unprecedented challenges, and these challenges had to be addressed while assessing the short-term and long-term effects of the vaccines [5]. The adoption of vaccines has been demonstrated to be challenging, and vaccinations alone have been shown to be insufficient to shift the pandemic from its acute to its chronic phase. Continuing unprecedented preventive measures and precautionary strategies should be included alongside vaccination campaigns, especially to avoid the long-term spread of the virus and the emergence of new variants [5]. In early 2021, different international health authorities declared various vaccines to have emergency use authorization [9,11,12]. The first vaccines that were introduced and approved in the Kingdom of Saudi Arabia were the ChAdOx1 nCoV-19 (also known as Oxford AstraZeneca) vaccine and the BNT162b2 (also known as Pfizer) vaccine [6]. The Food and Drug Authority approved the two vaccines. BNT162b2 is a nucleic acid vaccine based on a modified mRNA molecule that encodes for the spike protein of SARS-CoV-2. ChAdOx1 nCoV-19 is a modified viral-vector based vaccine derived from the chimpanzee adenovirus, ChAdOx1, and encodes the SARS-CoV-2 spike protein, BNT162b2ChAdOx1 nCoV-19 [13].

On 16 July 2021, the mRNA-1273 (also known as Moderna) vaccine was added to the list of approved vaccines in the Kingdom of Saudi Arabia [14]. Several mild to moderate side effects, such as headaches, pain, swelling, and redness at the injection site, as well as muscle and joint aches, were associated with the side effects of the COVID-19 vaccinations [9,15,16]. In the eastern region of Saudi Arabia, similar common side effects during the first and second dose were reported such as fatigue, headache, and fever. Unusual side effects that have been previously reported include palpitations and irregular menstruation [14,17]. Further side effects such as pain at the injection site, feeling tired, and headaches were reported in Riyadh (the capital city of Saudi Arabia) [18].

There is limited literature regarding the third dose vaccination’s side effects in the general population [19], and in the population of Saudi Arabia [20], in particular, the western region of Saudi Arabia. Describing the consequences following the third vaccination dose, also known as the booster vaccination, compared with the first and second vaccination doses will aid in enhancing the understanding of the safety profile of the third dose compared to the first and second dose of the COVID-19 vaccinations and improve the vaccination process against COVID-19 [21,22]. Thus, this study aims to evaluate the short-term adverse effects of the third COVID-19 vaccination dose compared with the first and second vaccination doses in the Kingdom of Saudi Arabia among participants in the western region.

## 2. Materials and Methods

An online survey was distributed using a Google form with dual language (Arabic and English) for a cross-sectional study and was distributed to participants who were vaccinated with the BNT162b2, ChAdOx1 nCoV-19 and mRNA-1273 vaccines. The side effects were reported following the participants’ vaccinations. The survey was distributed in the western region of the Kingdom of Saudi Arabia (mainly in Makkah, Taif, and Jeddah) between the period of 26 January and 8 March 2022. The survey was revised by all authors to provide feedback on the survey sections and recommend any edits if needed.

The online questionnaire was designed and distributed on social media platforms including WhatsApp and Twitter; emails were also circulated to public health and university staff. Following entry to the online questionnaire, participants were asked to carefully read the comprehensive explanation of the purpose of the study prior to giving consent on a compulsory electronic consent form, comprising the data for voluntary participation and anonymity. An e-mail address was generated to facilitate communication between the participants and study researchers. Upon completion of the online survey, the data was anonymously collected and stored in a safe file. The structured online questionnaire contained two sections: The first section aimed to explore the participants’ demographical information (gender, age, nationality, and education) alongside their SARS-CoV-2 infection status (chronic conditions, previous infection with COVID-19, consumed medications and antibiotics). The second section aimed to investigate the type of vaccine received, the side effects post vaccination (first, second, or third dose), the duration of the encountered side effects, and any analgesics consumed to reduce the severity of the side effects. In addition, on days 1, 2, 3, and 4 following immunizations, the participants were requested to illustrate the intensity degree of each symptom, ranging from mild to severe. Additionally, the participants were asked about the average timing of the onset of side effects. All the participants who declined to take part, or who were not vaccinated with three doses, and participants who received vaccines other than BNT162b2, -ChAdOx1 nCoV-19, or mRNA-1273 were excluded. The sample size was calculated through raosoft.com, which indicated 385 participants as a sufficient sample size to achieve a 5.35% margin of error and 95% confidence. 

### 2.1. Statistical Analysis

For the data, the statistical analysis was performed using Statistics Package Social Science (SPSS) Version 25 (IBM Armonk, New York, NY, USA). Descriptive statistics were used as qualitative data with a *p*-value ≤ 0.05 was considered significant. The descriptive statistics were expressed as qualitative data to compare the symptoms between the types of vaccines and their side effect. Using the Mann–Whitney U test, the researchers conducted dimensional comparisons between the groups described herein.

### 2.2. Ethical Approval

The committee of the Institutional Review Board (IRB) at Imam Mohammad ibn Saud Islamic University has reviewed and approved this research with project number 167-2021, dated 20 December 2021. The IRB-approved study was titled, ‘Evaluate the Side Effect Associated with Three Dosage of the Covid-19 Vaccine on Adults in western Region, Saudi Arabia: A Cross-Sectional Study’.

## 3. Results

### 3.1. Demographic Characteristics of the Participants and Medical History

In this study, 574 participants were involved, but 161 were eliminated, because they did not meet the inclusion criteria. Therefore, the final sample size was reduced to 413 participants. The majority of participants were between 18 and 25 years of age (n = 138, 33.4%), followed by 41 and 60 years (n = 119, 28.8%), then 31 and 40 years (n = 78, 18.8%), then 26 and 30 years (n = 70, 16.9%), and only eight (1.9%) participants were >60 years of age. In terms of nationalities, 344 (83.2%) were Saudi Arabian, while the remaining 69 (16.7%) were non-Saudi Arabian participants (Table 1). The applicants’ general history was summarized in Table 1, with 84.3% of participants declaring no clinical history, while 44 (10.7%) reported a chronic disease and 21 (5.1%) reported previous health problems.

### 3.2. Participants’ Side Effects per Dose

In Table 2, the side effects were reported for the first, second, and third doses of the COVID-19 vaccinations (ChAdOx1 nCoV-19, mRNA-1273, and BNT162b2).

**First doses**: Most participants received BNT162b2 BioNTech (71.4%), followed by Oxford-ChAdOx1 nCoV-19 (27.3%), and mRNA-1273 (0.7%) (Table 2). Nearly 38% of the participants experienced side effects the day after vaccination. In comparison, 43.6 % of participants showed adverse effects on the second and third days after vaccination. The most reported adverse effects among the trial participants were injection site pain (54%), followed by muscle and/or joint pain (36.3%), then fatigue and headache (35.1%). However, menstrual disorder, dizziness, vomiting, breathing congestion, chest pain, hair loss, and skin itching or rash were less commonly reported by the study participants (Table 2 and Table 3). A total of 74% of participants indicated that the severity of the side effects was mild or moderately severe; leaving only 11.9% who suffered from severe adverse side effects. Most participants consumed pain relief medication to reduce the side effects’ severity (64.9%). In contrast, only 16.9% of participants indicated that there were no adverse effects following the first vaccination.

**Second dose:** Most participants received BNT162b2 (78%), followed by ChAdOx1 nCoV-19 (18.2%), and mRNA-1273 (3.6%) (Table 2). Nearly 40.9% of the participants experienced side effects on the next vaccination day. A total of 40.4% of participants showed adverse effects on the second and third days after vaccination. In particular, the most reported adverse effects among the trial participants were pain at the injection site (57.6%), fatigue and headache (42.1%), and muscle and/or joint pain (32.9%). However, dizziness, menstrual disorder, vomiting, breathing problems, hair loss, chest pain, and skin rashes and itching were reported less frequently by study participants (Table 2 and Table 3). Only 13.8% of participants encountered severe side effects. However, 62% of the participants consumed some medication to reduce the severity of the side effects. Only 16.9% of participants indicated that there were no adverse effects following the second vaccination.

**Third dose:** Most participants were vaccinated with BNT162b2 (85.5%), followed by mRNA-1273 (10.1%), and ChAdOx1 nCoV-19 (4.4%) (Table 2). Almost 44.8% of the participants experienced side effects on the day after vaccination. In comparison, 38% of participants showed adverse effects on the second and third days after vaccination. In particular, the most reported adverse effects among the trial participants were injection site pain (55.4%), fatigue and headache (45.3%), and muscle and/or joint pain (32.4%). Less commonly and rarely reported side effects were menstrual disorder, vomiting, breathing congestion, hair loss, chest pain, and skin itching or rash. A total of 62.5% of participants received some medication to reduce the severity of the side effects.

Table 4 shows the differences in the severe symptoms depending on the type of COVID-19 vaccination. In fact, it was found that there were high significant differences (*p* value < 0.01) between those who received the BNT162b2 vaccine and those who took the ChAdOx1 nCoV-19 vaccine and mRNA-1273 vaccine in terms of the severity of symptoms after the first and second doses (*p* value of 0.0001 and 0.006, respectively). While there were no significant differences in the severity of symptoms after the third dose (*p* value of 0.867) (Table 4).

## 4. Discussion

Most countries have taken precautions to limit the spread of SARS-CoV-2 since the COVID-19 pandemic started in December 2019 [9,10]. Saudi Arabia was one of the earliest countries to initiate early immunization efforts after COVID-19 vaccination approval by the Saudi Ministry of Health and the World Health Organization [9]. At the start of 2021, a number of vaccine candidates were authorized for emergency use by several international health organizations [9,11,12,23]. Initially, Saudi Arabia authorized the use of BNT162b2, followed by the vaccine developed by Oxford AstraZeneca, ChAdOx1 nCoV-19 [9], then the mRNA-1273 vaccine [14]. Consequently, in this study, participants who received three doses of the COVID-19 vaccine in the western Saudi Arabian region were evaluated for any short-term adverse events of the third COVID-19 vaccination dose compared to the first and second vaccination doses. In this study, most participants received BNT162b2, followed by ChAdOx1 nCoV-19 vaccines. Nevertheless, it was found that more participants encountered severe side effects with ChAdOx1 nCoV-19 for the first and second doses compare to BNT162b2-BioNTech and mRNA-1273. Alghamdi et al. also demonstrated that the severity of the side effects from ChAdOx1 nCoV-19 was higher compared to BNT162b2 in both the first and second doses. The incidence of mild adverse events was 30.1%, and 29.7% for severe side effects following the administration of the ChAdOx1 vaccination [14]. In comparison, the majority of participants who received BNT162b2 vaccinations only experienced mild side effects (63.92%); only 7.68% experienced severe side effects. However, our findings suggest that the severity of ChAdOx1 side effects were not significantly different compared with those for BNT162b2.

Patients who received the third COVID-19 vaccination dose encountered side effects such as pain at the site of injection, fatigue and headache, which were closely similar in percentage to the side effects following the second dose. Indeed, the most common systemic and local side effects of the COVID-19 vaccination were fatigue and tenderness [24]. It was found that the most common three side effects in all three dosages were fatigue and headache, pain at the injection site, and muscle and joint pain. These side effects were mostly common despite the difference in vaccination type. In contrast, the rare side effects reported included vomiting, breathing congestion, a skin rash, chest pain, and a drop in sugar level, showed very similar percentages between patients for all three doses. Supporting our findings, it was previously reported that the most common side effects included discomfort at the injection site, fever, headache, fatigue, and flu-like symptoms, while sleepiness, difficulty breathing, and body aches were less prevalent [25].

Our results showed that the most reported level of severity of the side effects was moderate. Unlike previous findings, a mild level of severity of symptoms was outstanding [14,21]. The majority of the findings concluded that the severity of the COVID-19 vaccinations ranged between moderate and mild, which supports the safety profile for the COVID-19 vaccines approved by the Saudi Arabian Ministry of Health [22]. Most of the participants reported that each dose’s adverse effects lasted one—two days and took medication to decrease the severity of symptoms [25]. Similarly, the duration of symptoms after the first and second doses lasted for one—two days, which was highly reported previously [9,14,19].

The results demonstrated that there was a difference between genders in terms of the severity of symptoms for the first, second, and third doses, and the averages showed that the severity of symptoms was higher in females. This data supports previous research and indicates that females were considerably more likely than men to experience side effects following vaccination [14,15,20,26]. This might indicate that the Covid-19 vaccines function by stimulating the immune system, which can impact females more due to gender-based differences in the immune response, as observed in vaccines for diseases such as measles, mumps, and many others [24]. Furthermore, this may be attributable to hormonal variations. Since ACE2 expression is coded by the X chromosome, it is possible that males and females experience distinct patterns of its regulation [27]. In the past, it was revealed that women have lower levels of ACE2 expression in the lung, which led researchers to hypothesize that estrogen might decrease ACE2 expression [27]. Androgen also upregulates the mucosa-specific serine protease TMPRSS2, which helps viruses enter human host cells [28]. One of the enzymes that aids virus entry is TMPRSS2 [29]. This adds to the evidence that implies sex hormones may make it easier for males to become infected with COVID-19. Furthermore, several studies have shown that women develop stronger antibodies in response to infection and vaccination than men. For example, estrogens were shown to upregulate antibody development in mice, whereas testosterone suppressed antibody production. Furthermore, men with higher testosterone levels had a lower immune response to influenza vaccination than men with normal testosterone levels and women [30]. Several clinical trials have explored the application of hormone therapy to treat COVID-19 [31]. Because men are more likely to develop severe illness and die from the virus, researchers wondered if treating acute COVID-19 infection with female sex hormones could improve disease outcomes [6].

Furthermore, we found that there were statistically significant differences (*p* < 0.001) in the severity of symptoms between those who has taken analgesics to reduce the severity of their symptoms and those who did not after the three doses and found that the severity of the symptoms was greater in those who took analgesics. 

Our study is the first Saudi Arabian western region-based investigation of COVID-19 vaccination adverse effects; however, this study had a few limitations. These include variations in patient interpretation and tolerance levels, and the fact that the outcomes of the questionnaire, which was circulated in the western region of Saudi Arabia, were self-reported by vaccinated participants and have not been clinically validated by expert clinicians. Heterogeneity in participant responses may have been caused by using a subjective scale rather than an objective standard to classify the severity of symptoms, such as mild, moderate, or severe. In addition, when completing the survey more than five days after vaccination, the participants were susceptible to recall bias, which hindered the accuracy of their memories. In addition, the survey did not question the incidence of immediate allergic reaction after vaccination for three doses. In order to comprehend the relationship between risk factors and developing side effects, larger participant studies need to be performed to widely evaluate the side-effects severity difference of the third dose compared to the first and second doses, depending on the type of vaccine administered. Moreover, many confounding variables may influence the interpretation of the data.

## 5. Conclusions

In conclusion, Saudi Arabia was one of the first countries to begin the administration of booster vaccinations for the whole Saudi Arabian population. Our cross-sectional study was concentrated in the western region of Saudi Arabia to evaluate the severity of side-effects for the third dose in comparison with the first and second vaccination doses. We found that the third dose had higher severe frequency of side effects as described by patients, which was not dependent on the type of immunization vaccine. In addition, there were no significant differences between the type of side effects reported for the three doses, including the rare side effects encountered in our study. These results indicate that the incidence of severe side effects following the third dose vaccination is frequent, while there are no differences between side effects for the first, second, and third doses after vaccination. Follow-up studies on larger populations are needed to assess vaccine efficacy in controlling and preventing COVID-19 infections, as well as the long- and short-term side effects.

## Figures and Tables

**Table 1:** Overview of participant demographics and medical history.

Variables	Patient Number	Percent (%)
**Gender**		
Male	262	63.4
Female	151	36.6
**Age groups**		
18–25	138	33.4
26–30	70	9.4
31–40	78	34.4
41–60	119	29.1
>60	8	3.5
**Nationality**		
Saudi	344	83.3
Non-Saudi	69	16.7
**Education**		
Teacher	136	33.4
Student	111	26.8
Non-student/Unemployed	90	21.7
Other employment	76	18.4
**Health status**		
Good health	348	84.3
Health problem	21	5.1
Chronic disease	44	10.7
Diagnosed with coronavirus infection before you received the first dose of the coronavirus vaccine	41	17.3
Not diagnosed with coronavirus infection before you received the first dose of the coronavirus vaccine	196	82.7
Receiving antimicrobial agents	6	1.2
Not receiving antimicrobial agents	407	98.5
Taking any medications to treat any disease	96	23.2
Not taking any medications to treat any disease	317	76.8

**Table 2:** COVID-19 vaccination and the side effects encountered with BNT16b2, ChAdOx1 nCoV-19 and mRNA-1273.

	First Dose Number (%)	Second Dose Number (%)	Third Dose Number (%)
**Type of vaccine** BNT162b2 ChAdOx1 nCoV-19 mRNA-1273 Diagnosed with coronavirus infection before receiving the COVID-19 vaccination	295 (71.4)	322 (78)	353 (85.5)
	113 (27.3)	75 (18.2)	18 (4.4)
	3 (0.7) 41 (17.3)	15 (3.5) 0 (0)	42 (10.1) 46 (11.1)
**Common side effects after vaccination** Fatigue and/or headache Pain at the site of injection Muscle and/or joint pain High temperature and shivering Dizziness Menstrual disorder No side effects	145 (35.1)	174 (42.1)	187 (45.3)
	223 (54) 150 (36.3) 56 (13.6) 223 (54) 150 (36.3) 138 (33.4)	238 (57.6) 136 (32.9) 174 (42.1) 238 (57.6) 136 (32.9) 48 (29.5)	229 (55.4) 134 (32.4) 118 (28.6) 60 (14.5) 33 (8) 139 (33.7)
**Severity of side effects** Mild Moderate Severe			
	56 (13.6)	48 (11.6)	116 (28.1)
	51 (12.3)	44 (10.7)	157 (38)
	69 (16.9)	70 (16.94)	89 (21.5)
**When did you feel the side effects after the first dose of the coronavirus vaccine? ** First day Second day Third day	157 (38)	169 (40.9)	185 (44.8)
	180 (43.6) 15 (3.6)	167 (40.4) 13 (3.1)	157 (38) 16 (3.9)
**How long did the side effect last after vaccination?** One—two days Three days Four days or more	226 (54.7)	243 (58.8)	224 (54.2)
	97 (23.5) 36 (8.7)	77 (18.6) 34 (8.2)	77 (18.6) 56 (13.6)
**Any medication taken?** Medication taken to reduce the severity of the side effects. No medication taken to reduce the severity of the side effects.	268 (64.9) 145 (35.1)	257 (62.2) 156 (37.8)	258 (62.5) 155 (37.5)

**Table 3:** Less common side effects after vaccine.

Less Common Side Effect	First Dose Number (%)	Second Dose Number (%)	Third Dose Number (%)
Vomiting	8 (1.9)	15 (3.6)	22 (5.3)
Breathing congestion	20 (4.8)	18 (4.4)	25 (6.1)
Skin itching or rash	20 (4.8)	21 (5.1)	25 (6.1)
Drop in sugar level	1 (0.2)	0 (0)	1 (0.2)
Chest pain	2 (0.5)	2 (0.5)	2 (0.5)

**Table 4:** The significance of symptoms’ severity depending on the type of vaccination for the three doses.

	Type of COVID-19 Vaccine	N	Mean Rank	Kruskal–Wallis H	*p* Value
Side effects’ severity following the first dose	BNT162b2	295	192.57	17.746	0.0001
	ChAdOx1 nCoV-19	113	244.66		
	mRNA-1273	5	207.50		
	Total	413			
Side effects’ severity following the second dose	BNT162b2	322	205.65	10.115	0.006
	ChAdOx1 nCoV-19	75	194.64		
	mRNA-1273	16	292.06		
	Total	413			
Side effects’ severity following the third dose	BNT162b2	353	205.93	0.286	0.867
	ChAdOx1 nCoV-19	18	219.31		
	mRNA-1273	42	210.71		
	Total	413			

## Data Availability

The data used for the findings of the current study are available upon request from the corresponding author.
